# Potential mechanisms of attenuation for rifampicin-passaged strains of *Flavobacterium psychrophilum*

**DOI:** 10.1186/s12866-015-0518-1

**Published:** 2015-09-16

**Authors:** Karol Gliniewicz, Mark Wildung, Lisa H. Orfe, Gregory D. Wiens, Kenneth D. Cain, Kevin K. Lahmers, Kevin R. Snekvik, Douglas R. Call

**Affiliations:** Department of Veterinary Microbiology and Pathology, Washington State University, Pullman, WA USA; Molecular Biology and Genomics Core, Washington State University, Pullman, WA USA; USDA-ARS-National Center for Cool and Cold Water Aquaculture, Leetown, WV USA; Department of Fish and Wildlife Resources, University of Idaho, Moscow, ID USA; Department of Biomedical Sciences and Pathobiology, Virginia Polytechnic Institute and State University, Blacksburg, VA USA; Washington Animal Disease Diagnostic Laboratory, Washington State University, Pullman, WA USA; Paul G. Allen School for Global Animal Health, Washington State University, Pullman, WA USA; Present address: Department of Biological Sciences, University of Idaho, Moscow, Idaho USA

**Keywords:** Rifampicin, Attenuation, *Flavobacterium psychrophilum*, SNP, Methylome

## Abstract

**Background:**

*Flavobacterium psychrophilum* is the etiologic agent of bacterial coldwater disease in salmonids. Earlier research showed that a rifampicin-passaged strain of *F. psychrophilum* (CSF 259-93B.17) caused no disease in rainbow trout (*Oncorhynchus mykiss*, Walbaum) while inducing a protective immune response against challenge with the virulent CSF 259–93 strain. We hypothesized that rifampicin passage leads to an accumulation of genomic mutations that, by chance, reduce virulence. To assess the pattern of phenotypic and genotypic changes associated with passage, we examined proteomic, LPS and single-nucleotide polymorphism (SNP) differences for two *F. psychrophilum* strains (CSF 259–93 and THC 02–90) that were passaged with and without rifampicin selection.

**Results:**

Rifampicin resistance was conveyed by expected mutations in *rpoB*, although affecting different DNA bases depending on the strain. One rifampicin-passaged CSF 259–93 strain (CR) was attenuated (4 % mortality) in challenged fish, but only accumulated eight nonsynonymous SNPs compared to the parent strain. A CSF 259–93 strain passaged without rifampicin (CN) accumulated five nonsynonymous SNPs and was partially attenuated (28 % mortality) compared to the parent strain (54.5 % mortality). In contrast, there were no significant change in fish mortalities among THC 02–90 wild-type and passaged strains, despite numerous SNPs accumulated during passage with (*n* = 174) and without rifampicin (*n* = 126). While only three missense SNPs were associated with attenuation, a Ser492Phe *rpoB* mutation in the CR strain may contribute to further attenuation. All strains except CR retained a gliding motility phenotype. Few proteomic differences were observed by 2D SDS-PAGE and there were no apparent changes in LPS between strains. Comparative methylome analysis of two strains (CR and TR) identified no shared methylation motifs for these two strains.

**Conclusion:**

Multiple genomic changes arose during passage experiments with rifampicin selection pressure. Consistent with our hypothesis, unique strain-specific mutations were detected for the fully attenuated (CR), partially attenuated (CN) and another fully attenuated strain (B17).

**Electronic supplementary material:**

The online version of this article (doi:10.1186/s12866-015-0518-1) contains supplementary material, which is available to authorized users.

## Background

Serial passage of bacteria with exposure to rifampicin may result in rifampicin-resistant microorganisms that may be useful as live-attenuated vaccines [1]. Rifampicin (Rif) is a potent, broad-spectrum antibiotic from the rifamycin group that inhibits the β-subunit of prokaryotic DNA-dependent RNA polymerase (RNAP). The antibiotic acts by directly blocking elongation of mRNA transcripts and drug resistance is normally conferred by point mutations in *rpoB* gene that encodes the β-subunit of the RNAP [2–5], although alternative mechanisms of rifampicin resistance have been described [6]. Recently, rifampicin passage was used to generate live-attenuated vaccines against a number of bacterial diseases of fish including columnaris disease, edwardsiellosis, enteric septicemia of catfish and motile aeromonad septicemia [7–12] or brucellosis in cattle [1]. Similarly, a live-attenuated strain CSF259-93B.17 of *F. psychrophilum* was developed by passage with rifampicin and infection with this strain induces a protective immune response in rainbow trout (*Oncorhynchus mykiss*, Walbaum) against challenge with the virulent parent CSF 259–93 strain [13]. Further analysis of the CSF259-93B.17 (B17) strain revealed a point mutation in the *rpoB* gene and numerous proteomic changes as compared to the parent strain [14].

Although the method of passaging pathogens with rifampicin has been successfully used to generate live vaccines for more than two decades, the mechanism of attenuation from this procedure remains unknown. That is, it is not clear if loss of virulence is directly associated with point mutations within the *rpoB* gene that confer resistance to rifampicin, or if accumulation of random mutations resulting from repeated passages with antibiotic selection pressure lead to attenuation, or perhaps a combination of both [9, 15–18]. This is an important question because knowing the mechanisms of attenuation provides information to better assess the likelihood that an attenuated strain might revert to a virulent phenotype in the future. Furthermore, knowing the mechanisms involved could lead to more efficient strategies to develop live-attenuated strains that do not rely on random chance.

In this study we passaged two pathogenic strains of *F. psychrophilum*, CSF 259–93 and THC 02–90, on media with and without rifampicin, and applied next-generation genome sequencing techniques and other methods to analyze changes associated with these culture conditions. The choice of these two strains was based on the fact that they belong to two distinct genetic lineages of *F. psychrophilum* [19], and that both are highly virulent to salmonids. These characteristics make these strains suitable for bacterial challenge in our rainbow trout model for assessment of attenuation of passaged strains [13, 19].

## Results

### Growth comparison

**Fig. 1:**
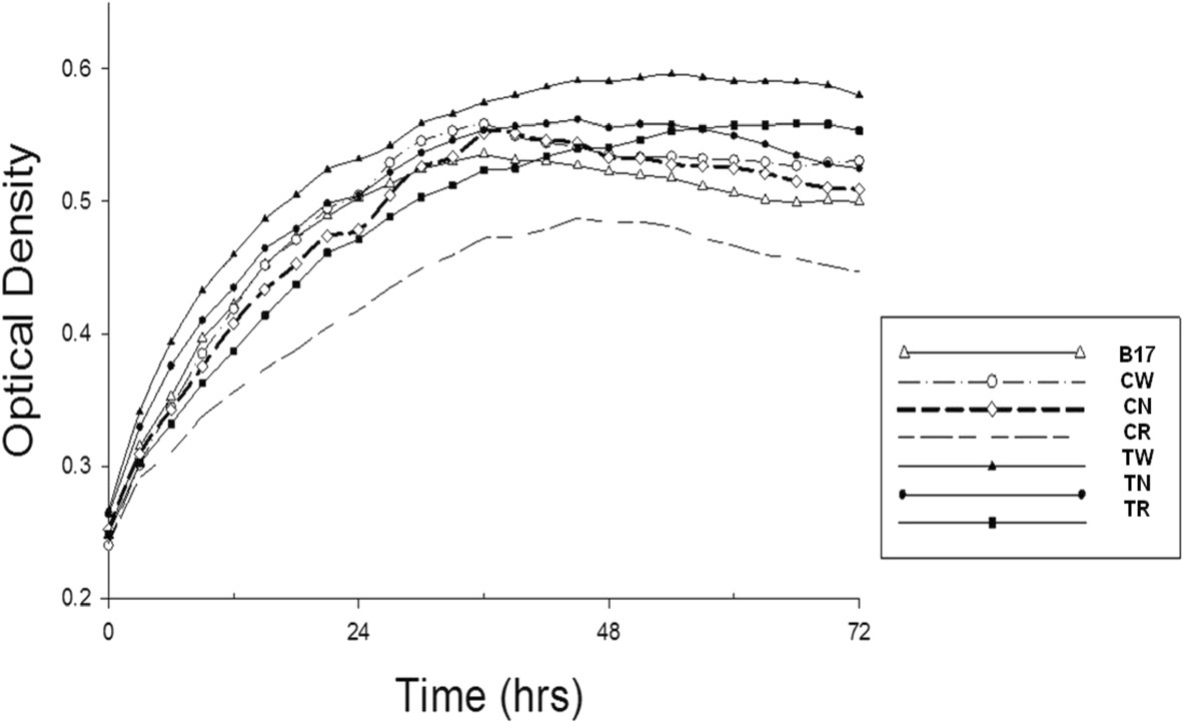
Growth curves of *F. psychrophilum* CSF 259–93 and THC 02–90 parent and passaged strains. Independent triplicates of each strain were grown statically in TYES broth at 16 °C with optical density measurements at 450–580 nm preceded by brief shacking. Abbreviations used: CW - *F. psychrophilum* CSF 259–93 parent strain, CN and CR – CSF 259–93 passaged 17 times with no and with rifampicin, respectively; B17 – rifampicin attenuated *F. psychrophilum* CSF 259-93B.17 strain [13]; TW - THC 02–90 parent strain and TN and TR – THC 02–90 passaged for 17 times with and without rifampicin, respectively

Growth kinetics of the parent *F. psychrophilum* strains (CSF 259–93 and THC 02–90) and strains passaged with and without rifampicin were determined in TYES broth at 16 °C and assessed with endpoint optical density measurements and the area-under-the-curve (AUC) comparisons (Fig. 1). In general, the parental strains (CW and TW) grew slightly faster than their passaged counterparts, but only the CR strain grew significantly slower when compared to its parental CW strain (*P* < 0.005). For THC 02–90 strains, even though the wild-type TW strain exhibited slightly better growth, there was no statistical difference between growth rates of TW, TN and TR strains for both endpoint OD and AUC measurements. All measurements of growth included three independent biological replicates per strain.

### Cell and colony morphology

**Fig. 2:**
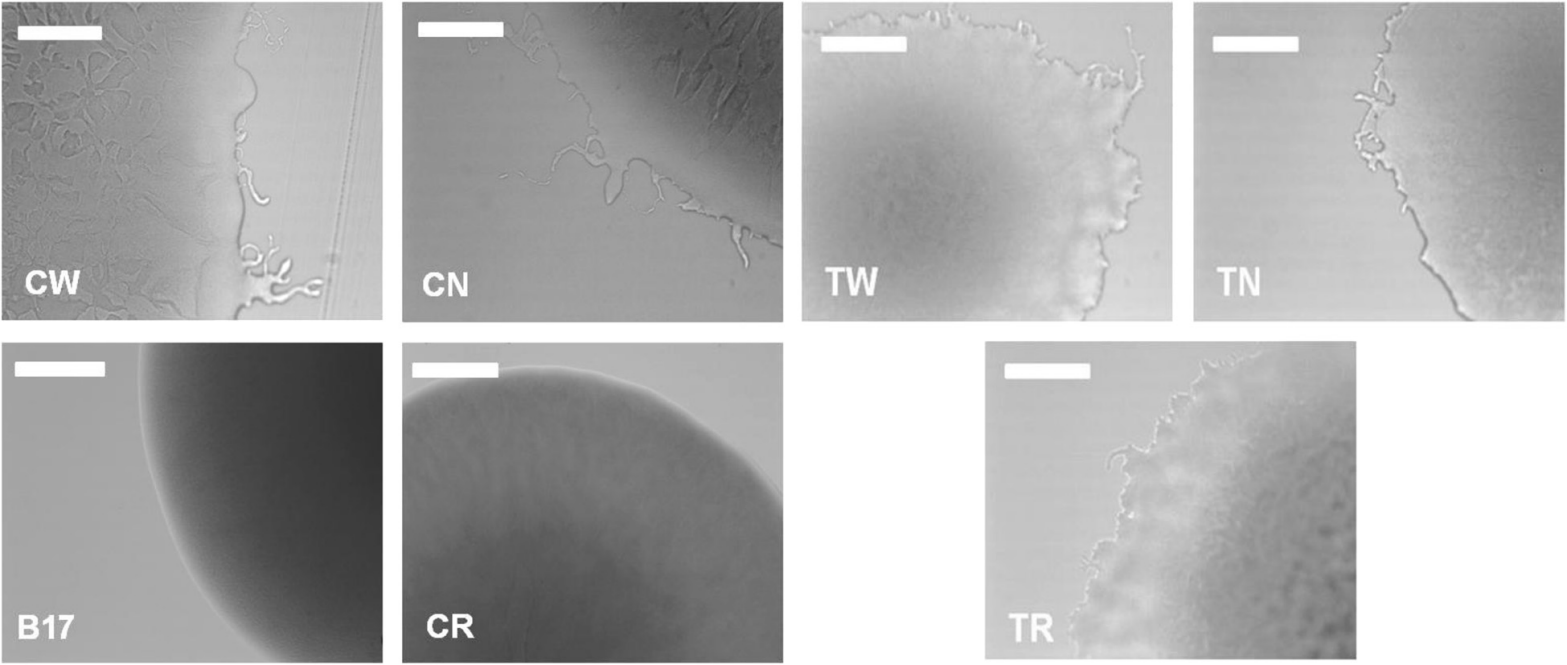
Light micrographs showing representative colony edges after 3-day incubation at 16 °C on gliding motility agar. CW – wild-type CSF 259.93; CN – CSF 259–93 passaged without rifampicin; CR– CSF 259–93 passaged with rifampicin; B17 – attenuated CSF 259.93B.17; TW – wild-type THC 02–90; TN – THC 02–90 passaged without rifampicin; TR – THC 02–90 passaged with rifampicin; white bars indicate 200 μm scale

Gram staining and microscopy showed no apparent differences in size or shape of cells from the six *F. psychrophilum* strains (data not shown). When grown on TYES agar the CR strain exhibited reduced yellow pigmentation compared with the CW and CN strains (data not shown). Additionally, when cultured on gliding motility agar the CR colonies showed decreased spreading indicative of impaired gliding motility (Fig. 2). There was no obvious motility impairment for the remaining strains and the TR strain appeared to be the most motile. From a qualitative perspective, all three THC 02–90 strains appeared to be more motile than the CSF 259–93 strains.

### rpoB mutations

Analysis of *rpoB* mutations from rifampicin resistant CR, TR and B17 strains obtained through genome sequencing, validated later using sequencing of PCR amplified *rpoB* (data not shown), revealed the presence of point mutations that are distinctive for these three strains. While the completely attenuated B17 strain harbors a Gln474Arg mutation, the *rpoB* of the CR strain is changed at Ser492Phe. The *rpoB* gene of the TR strain has a double mutation with Asp477Tyr and Pro496Ser substitutions (numbering based on NCBI reference sequence NC_009613.3).

### Carbohydrate and protein characterization

**Fig. 3:**
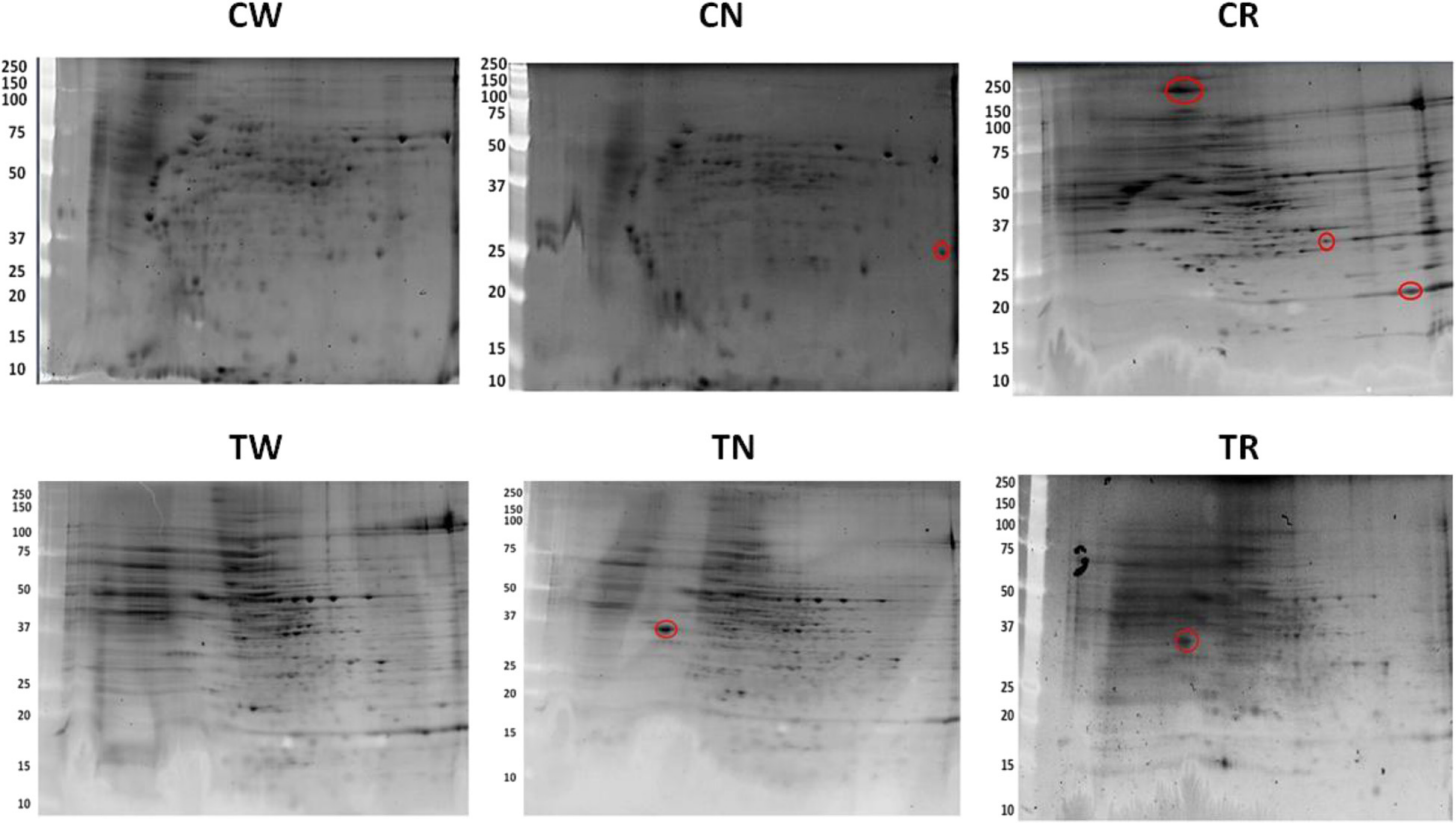
2D PAGE analysis of whole-cell lysates from the *F. psychrophilum* strains. Proteins were stained with SyPro Ruby gel stain and molecular weight markers (kDa) are indicated on the left of each gel. Red ovals designate proteins reproducibly greater abundance as compared to the respective (CW or TW) parent strain. CW – wild-type CSF 259.93; CN – CSF 259–93 passaged without rifampicin; CR– CSF 259–93 passaged with rifampicin; B17 – attenuated CSF 259.93B.17; TW – wild-type THC 02–90; TN – THC 02–90 passaged without rifampicin; TR – THC 02–90 passaged with rifampicin

Bacterial proteinase-K digested carbohydrate extractions and whole-cell lysates were prepared from each strain and analyzed by SDS-PAGE and 2D PAGE, respectively. There were no visible differences in LPS banding patterns among the six strains, although there were visual differences in band intensities (Additional file 1: Figure S1). Proteomic analysis of the CN strain showed increased synthesis of a protein with a molecular mass of approximately 25 kDa as compared to its parent strain. Additionally, whole-cell lysates the CR strain revealed that synthesis of three proteins of 20, 35 and 200 kDa was qualitatively increased. Both TN and TR appeared to have increased expression of a similar protein with approximate molecular mass of 35 kDa when compared to their parent TW strain (Fig. 3).

### Analysis of single-nucleotide polymorphisms

Initial 454 sequencing of a CW and B17 strain yielded 179,155 reads totaling 63.1 Mb for the CW strain. When compared to the CSF 259–93 reference genome, 99.3 % of the CW reads assembled into 109 contigs covering 2,769,031 bp with mean length of contig size of 69.8 kb. Most (99.8 %) of bases were Q40+ quality and an additional 17,852 bp were assembled into 66 shorter contigs. Sequencing of the B17 strain yielded 449,264 reads totaling 167 Mb, and assembly to reference sequence resulted in 98.9 % bases assembled into 107 contigs covering 2,779,773 bp. Most (99.6 %) of the base pairs were Q40+ and the mean contig size was 70.7 kb. An additional 25,684 bp were included in 60 shorter contigs. Results of IonTorrent sequencing of CW, CN, CR, TW, TN and TR strains are shown in Table 1.

**Table 1:** Summary of output from IonTorrent and 454 sequencing for seven *F. psychrophilum* strains

Sample	Mb total^a^	Number of reads (in millions)	Mean read length (in bp)
CSF 259–93 wild-type (CW)^b^	603	2.46	245
CSF 259–93 passaged w/o Rif (CN)^b^	346	1.37	254
CSF 259–93 passaged with Rif (CR)^b^	772	2.97	261
THC 02–90 wild-type (TW)^b^	620	2.38	260
THC 02–90 passaged w/o Rif (TN)^b^	676	2.78	244
THC 02–90 passaged with Rif (TR)^b^	666	2.78	240
CSF 259–93 wild-type (CW)^c^	63.1	0.18	N/A
CSF 259-93B.17 (B17)^c^	167	0.45	N/A

^a^Mb total = total number of mega bases of DNA sequences

^b^Results from Ion Torrent sequencing, 316 chip

^c^Results from 454 sequencing

We based our SNP analysis on polymorphisms with read frequency ≥80 % and we focused on mutations that led to predicted nonsynonymous amino acid changes (Table 2). Our wild-type CSF 259–93 strain exhibited 19 SNPs leading to nonsynonymous amino acid changes when compared to the reference genome of reported for CSF 259–93 (GenBank Accession number: CP007627.1). These 19 SNPs included codon changes in 14 genes leading to nonsynonymous amino acid changes (Table 3) and may reflect mutations that have accumulated over years of passage in different labs although these two sequenced strains retain their virulence against rainbow trout. Analysis of the B17 strain’s genome after 454 sequencing revealed SNPs resulting in 14 nonsynonymous amino acid changes in codons of 14 genes when compared to its parental CW strain (Table 4). Genomes from CN and CR strains accumulated 5 and 8 SNPs, respectively, when compared to their parental CSF 259–93 strain (CW). These SNPs led to missense mutations in codons of 4 genes in the CN strain (Table 5), and 7 in case of CR (Table 6). After comparison with the genome of their parental THC 02–90 strain (TW), TN and TR strains displayed numerous differences. The TN strain accumulated 126 SNPs resulting in missense mutations in codons of 48 genes (Additional file 1: Table S1), and in the TR strain 174 SNPs led to 64 genes with codons leading to nonsynonymous amino acid substitutions (Additional file 1: Table S2). Interestingly, despite the large number of missense mutations in the THC 02–90 passaged strains there was no evidence for commensurate changes in the proteome for these strains relative to the wild-type proteome (Fig. 3).

**Table 2:** Single nucleotide polymorphisms (SNPs) in *F. psychrophilum* strains used in our experiments

		NONSYNONYMOUS SNPs							
SYNONYMOUS SNPs		CW	CN	CR	TW	TN	TR	ERGO	B17
	CW	n.a.	5	8	85	113	123	19	14
	CN	32	n.a.	3	105	141	117	25	N.D.
	CR	34	33	n.a.	113	158	108	28	N.D.
	TW	161	170	174	n.a.	126	174	100	N.D.
	TN	233	142	146	177	n.a.	48	102	N.D.
	TR	278	287	291	257	180	n.a.	132	N.D.
	ERGO	37	75	78	206	208	238	n.a.	33
	B17	45	N.D.	N.D.	N.D.	N.D.	N.D.	50	n.a.

*CW* wild type CSF 259–93, *CN* CSF 259–93 passaged without Rif, *CR* CSF 259–93 passaged with Rif, *TW* wild type THC 02–90, *TN* THC 02–90 passaged without Rif, THC 02–90 passaged with Rif, B17 – CSF 259-93B.17

*N.D.* not determined due to these genomes being sequenced at different times and by different methods

n.a. = not applicable

**Table 3:** Analysis of single nucleotide polymorphisms (SNPs) from *F. psychrophilum* CSF 259–93 used in our laboratory

Reference position^a^	Allele frequency (in %)^b^	SNP^c^	Coverage^d^	Annotation^e^	Amino acid change^e^	Gene/Protein^f^
721449	100	C > A	72	FPSM_00632	Gly111Trp	Hypothetical protein
742016	100	A > G	70	FPSM_00646	Ser615Gly	Type II restriction-modification system methylation subunit
899151	100	C > A	84	FPSM_00800	Phe215Leu	RmuC family protein
899152	100	G > A	92	FPSM_00800	Gly216Glu	
921140	96.7	C > A	90	FPSM_00823	Pro254Thr	Hypothetical protein (similar to FP1973-GldK gliding motility protein)
1257208	100	C > G	98	FPSM_01126	Trp148Ser	Putative membrane spanning protein
1333946	97.7	A > T	43	FPSM_01204	Phe507Ile	Glucose/galactose transporter
1334321	98.7	A > C	78	FPSM_01204	Tyr382Asp	
1387849	95.1	T > G	61	FPSM_01258	Leu133Arg	Phage related protein
1387851	100	T > G	99	FPSM_01258	Tyr134Asn	
1537329	98.7	C > G	75	FPSM_01388	Arg547Thr	TonB-dependent outer membrane receptor
1630279	92	A > C	25	FPSM_01473	Thr160Pro	Transposase
1664395	100	C > G	81	FPSM_01493	Val213Leu	Cation efflux system plasma membrane protein (similar to FP0880 – probable multidrug resistance protein precursor AcrB/D/F family)
1664410	99	G > C	96	FPSM_01493	Pro208Ala	
1970401	97.4	T > C	116	FPSM_01788	Ser383Gly	Von Willebrand factor type A domain protein (FP0550 – *yfbK* probable outer membrane protein precursor)
1970443	100	C > T	122	FPSM_01788	Asp369Asn	
2066236	100	C > A	76	FPSM_01870	His300Asn	Hypothetical protein
2514049	98.5	C > G	130	FPSM_02290	Leu293Val	3-oxoacyl-(acyl carrier-protein) synthase III (FP1374 *fabH1*)
2737510	100	C > G	119	FPSM_02485	Leu163Phe	tRNA pseudouridine synthase B (FP2357 *truB*)

^a^Reference position (in bp) for reference sequence (listed under annotation field) are based on CSF 259–93 sequence from ERGO-Integrated genomics

^b^Allele frequency refers to the proportion of sequences showing a given single-nucleotide polymorphism (SNP) at the started reference position

^c^Shows the SNP change represents the change from nucleotide X to Y (X > Y) at the reference position

^d^Coverage refers to the total number of sequencing reads that align to each base within the sample DNA

^e^Predicted amino acid change for the identified SNP

^f^Annotation shows the name and putative function for the identified gene

**Table 4:** Single nucleotide polymorphisms (SNPs) from *F. psychrophilum* CSF 259-93B.17 strain

Reference position^a^	Allele frequency (in %)^b^	SNP^c^	Coverage^d^	Annotation^e^	Amino acid change^e^	Gene/Protein^f^
43576	100	A > T	5	FPSM_00026	Met1Leu	Multimodular transpeptidase-transglycosylase PBP 1A
155877	100	A > T	43	FPSM_00129	Leu71Phe	Putative exported protein
283611	80	A > T	10	FPSM_00250	Asn522Ile	Amino acid permease
325469	100	T > A	4	FPSM_00287	Met1Leu	Thioredoxin
488182	100	A > C	4	FPSM_00425	Gln466Pro	DNA primase
514297	86.4	C > T	162	FPSM_00445	Ala11Thr	Malonyl-CoA-[acyl-carrier-protein] transacylase
548737	100	A > T	5	FPSM_00479	Met1Leu	Ribosome recycling factor (RRF)
846079	100	T > A	4	FPSM_00743	Phe98Tyr	Putative membrane spanning protein
856484	85.7	T > A	7	FPSM_00754	Met13Leu	Hypothetical protein
1334056	100	A > C	39	FPSM_01204	Phe470Cys	Glucose/galactose transporter
1480580	80	A > T	5	FPSM_01337	Asn41Ile	Hypothetical protein
1498680	100	T > A	4	FPSM_01353	Asn123Ile	Phosphoribosyl-ATP cyclohydrolase
1522077	80	T > A	5	FPSM_01375	Met1Leu	Protein translation elongation factor P (EF-P)
1537329	100	C > G	51	FPSM_01388	Arg547Thr	TonB-dependent outer membrane receptor

^a^Reference position (in bp) for reference sequence (listed under annotation field) are based on CSF 259–93 sequence from ERGO-Integrated genomics

^b^Allele frequency refers to the proportion of sequences showing a given single-nucleotide polymorphism (SNP) at the started reference position

^c^Shows the SNP change represents the change from nucleotide X to Y (X > Y) at the reference position

^d^Coverage refers to the total number of sequencing reads that align to each base within the sample DNA

^e^Predicted amino acid change for the identified SNP

^f^Annotation shows the name and putative function for the identified gene. Annotation is presented from the CSF 259–93 sequence from ERGO-Integrated genomics

**Table 5:** Single nucleotide polymorphisms (SNPs) from *F. psychrophilum* CSF 259–93 strain passaged with no rifampicin (CN)

Reference position^a^	Allele frequency (in %)^b^	SNP^c^	Coverage^d^	Annotation^e^	Amino acid change^e^	Gene/Protein^f^
575838	100	C > G	71	FPSM_00502	Val638Leu	Ribonucleoside-diphosphate reductase large chain (*nrdA*)
824811	94	A > G	50	FPSM_00723	Ile62Thr	GTP-binding protein YihA
2606362	100	G > T	25	FPSM_02361	Trp4Leu	Putative membrane spanning protein
2606399	95	A > T	20	FPSM_02361	Leu16Phe	
2718910	100	T > C	83	FPSM_02464	Tyr418Cys	Di-tripeptide transporter

^a^Reference position (in bp) for reference sequence (listed under annotation field) are based on CSF 259–93 sequence from ERGO-Integrated genomics

^b^Allele frequency refers to the proportion of sequences showing a given single-nucleotide polymorphism (SNP) at the started reference position

^c^Shows the SNP change represents the change from nucleotide X to Y (X > Y) at the reference position

^d^Coverage refers to the total number of sequencing reads that align to each base within the sample DNA

^e^Predicted amino acid change for the identified SNP

^f^Annotation shows the name and putative function for the identified gene. Annotation is presented from the CSF 259–93 sequence from ERGO-Integrated genomics

**Table 6:** Single nucleotide polymorphisms (SNPs) from *F. psychrophilum* CSF 259–93 strain passaged with rifampicin (CR)

Reference position^a^	Allele frequency (in %)^b^	SNP^c^	Coverage^d^	Annotation^e^	Amino acid change^e^	Gene/Protein^f^
575838	100	C > G	71	FPSM_00502	Val638Leu	Ribonucleoside-diphosphate reductase large chain (*nrdA*)
824811	94	A > G	50	FPSM_00723	Ile62Thr	GTP-binding protein YihA
1327825	100	G > A	138	FPSM_01200	Asp275Val	Hypothetical protein
2111614	81.9	C > A	144	FPSM_01917	Thr365Lys	Phytoene desaturase (*crtI*)
2319546	98.8	G > A	167	FPSM_02089	Ser492Phe	DNA-directed RNA polymerase beta chain RpoB
2606236	100	G > T	73	FPSM_02361	Trp4Leu	Putative membrane spanning protein
2606399	100	A > T	65	FPSM_02361	Leu16Phe	
2788973	97.1	T > A	105	FPSM_02533	Ile35Phe	Transcriptional regulator, TetR family

^a^Reference position (in bp) for reference sequence (listed under annotation field) are based on CSF 259–93 sequence from ERGO-Integrated genomics

^b^Allele frequency refers to the proportion of sequences showing a given single-nucleotide polymorphism (SNP) at the started reference position

^c^Shows the SNP change represents the change from nucleotide X to Y (X > Y) at the reference position

^d^Coverage refers to the total number of sequencing reads that align to each base within the sample DNA

^e^Predicted amino acid change for the identified SNP

^f^Annotation shows the name and putative function for the identified gene. Annotation is presented from the CSF 259–93 sequence from ERGO-Integrated genomics

### DNA methylation analysis

We used Allora EC assemblies from the Pacific Biosciences sequencer to evaluate different DNA methylation patterns for CR and TR strain. RS ALLORA assembly EC.1 protocol of SMRT portal version 1.3.3 was used. Reads shorter than 100 bp and with quality below 0.85 were discarded. Postfiltered reads for the CR strain consisted of 385 Mb in 107,567 reads. The reads had an average length of 3581 bp and an average quality of 0.873. 100 % of the reads were assembled into 7 contigs with a sum of 2.27 Mb, a max contig size of 908,349 and an N50 of 511,868. Postfiltered reads for the TR strain had an average quality of 0.877 and totaled 449 Mb in 124,950 reads averaging 3590 bp. One hundred percent of the reads assembled into 7 contigs with a sum of 2.26 Mb and a max contig size of 710,139 bp and an N50 of 371,120 bp. DNA methylation was detected on both 7 contig assemblies and the corresponding motifs were identified with RS Modification and Motif Analysis.1 protocol of SMRT portal version 1.3.3.

Upon release of SMRT portal version 2.0.1 the existing PacBio data was reassembled using the RS HGAP Assembly 1 protocol. For the strain CR, less strict filtering allowed 830 Mb of data to enter the assembly with an average read length of 3658 bp and average quality of 0.847. Preassembly yield was 0.73 on a self-calculated minimum seed read size of 8038 bp, and generated 54 Mb in 7385 pre-assembled reads with an N50 of 8649 bp. The Celera assembler component of the HGAP algorithm assembled the pre-assembled reads into a single contig. Quiver was used to polish the assembly and found consensus concordance of 99.987 % with uniform 210× coverage and a polished contig size of 2,905,139 bp. For the strain TR, 927 Mb of data entered the assembly with an average read length of 3720 bp and average quality of 0.849. Preassembly yield was 0.73 on a self-calculated minimum seed read size of 8480 bp, and generated 54.8 Mb in 7137 pre-assembled reads with an N50 of 8987 bp. HGAP assembled the pre-assembled reads into two contigs. Quiver was used to polish the assembly and found consensus concordance of 99.991 % with uniform 232× coverage and polished contig sizes of 2,810,854 bp and 18,318 bp.

Genome wide methylation was detected by mapping the Pacific Biosciences reads onto the HGAP contigs and the corresponding modification motifs were identified using the RS Modification and Motif Analysis.1 protocol of SMRT portal version 2.0.1. Comparison of the CR and TR modifications resulted in identification of 8 DNA motifs in the CR and 16 in the TR that exhibited different methylation (Additional file 1: Table S3 and S4). No motifs were shared between these two strains.

### Assessment of attenuation

**Fig. 4:**
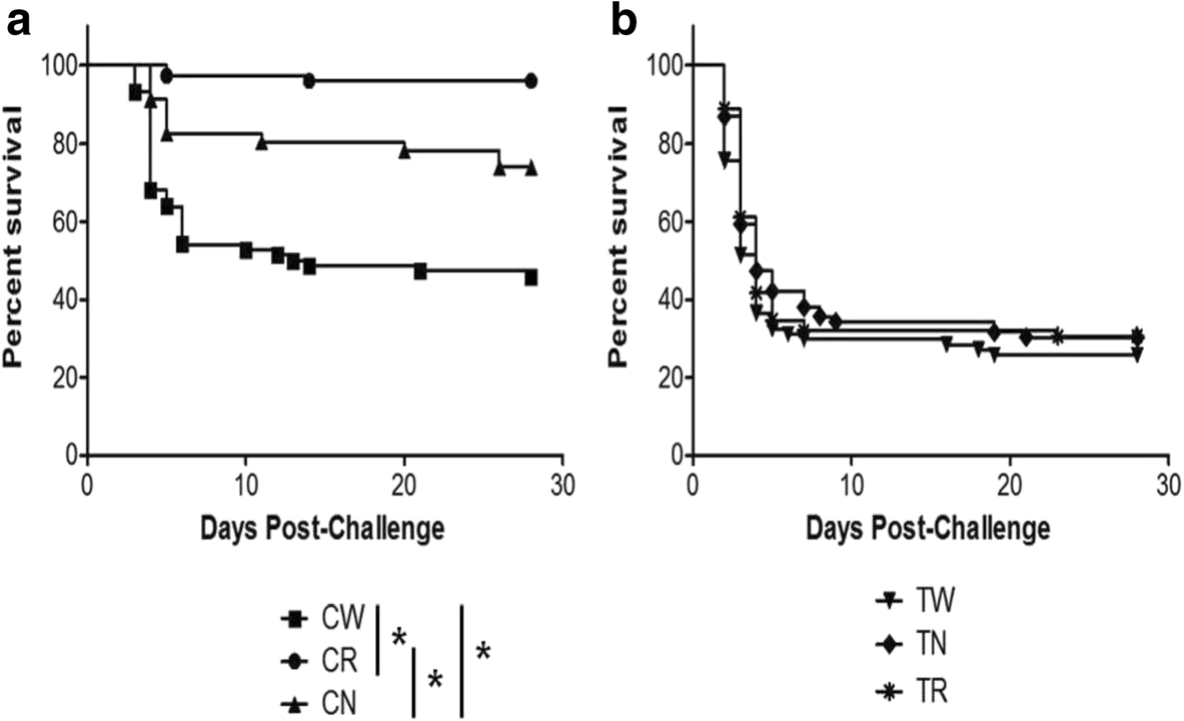
Percent survival curves of rainbow trout (*Oncorhynchus mykiss*, Walbaum) fry challenged with different *F. psychrophilum* strains. **a** Challenges with different CSF 259–93 strains with (panel **b**). showing comparisons between different THC 02–90 strains. Statistically significant values (*P* < 0.005) are indicated by asterisks. CW – wild-type CSF 259.93; CN – CSF 259–93 passaged without rifampicin; CR– CSF 259–93 passaged with rifampicin; B17 – attenuated CSF 259.93B.17; TW – wild-type THC 02–90; TN – THC 02–90 passaged without rifampicin; TR – THC 02–90 passaged with rifampicin

Challenge experiments with the CN strain demonstrated partial loss of virulence with cumulative percent mortality (CPM) of 28.1 % (standard error of the mean (SEM) ± 2.0 %) (Fig. 4). The CR strain appears to be almost completely attenuated inflicting only 4 % mortality (SEM ± 2.3 %). CPM of fish challenged with the wild-type CSF 259–93 strain (CW) reached 54.5 % (SEM ± 6.9 %). Log-rank analysis of mortality patterns (Fig. 4a) demonstrated that observed changes in virulence were statistically significant for survival in CN treatment compared to CW (*P* = 0.002) and for survival of CR group compared to CW (*P* < 0.0001). Changes in virulence between CN and CR were also statistically significant (*P =* 0.0003). Cumulative percent mortalities in case of TW, TN and TR were 74.3 % (SEM ± 3.5 %), 69.7 % (SEM ± 1.2 %) and 69.4 % (SEM ± 2.9 %), respectively (Fig. 4b). Log-rank analysis among the three THC 02–90 groups indicated no statistically significant differences in CPM values (TW vs. TR *P* = 0.27, TN vs. TR *P* = 0.27 and TN vs. TR *P* = 0.97). There was no mortality in PBS mock infected group. For all mortalities that we tested, culture of the kidney, liver and spleen tissue confirmed the presence of yellow-pigmented bacterial colonies that resembled typical morphology of *F. psychrophilum*.

## Discussion

Repeated laboratory passage of pathogens in the presence of rifampicin has been used to generate live-attenuated vaccines [1, 7–12], but the mechanism of rifampicin-induced attenuation remains unknown. There are a variety of potential confounding factors with this type of passage experiment that make cause-and-effect interpretations challenging. Passage can produce changes in global protein expression profiles [12, 13] or altered lipopolysaccharide (LPS) biosynthesis/colony roughness [7]. These changes could be attributed to altered activity of RpoB in the Rif resistant DNA-dependent RNA polymerase (RNAP) present in rifampicin resistant bacteria [14]. That is, the presence and activity of the mutated *rpoB* is one hypothesis for the mechanism of attenuation. Alternatively, rifampicin-associated attenuation may result from accumulation of spontaneous mutations acquired in the course of *in vitro* passaging. Serial passage of a pathogenic bacterium without any antibiotics can also induce changes in LPS profile and colony roughness, which have been correlated with attenuation [18]. This finding is consistent with the role of random mutations in the attenuation process, although the rate of mutation may differ depending on the stress imposed on the passaged microorganism [20, 21]. For example, for the current study the two strains that were passaged with rifampicin, CR and TR, accumulated more SNPs compared to their matched strains that were passaged without the antibiotic (CN and TN, 33.3 and 29.4 % respectively).

In the present study, passage did not affect in vitro growth characteristics except for the CR strain, which was compromised to some extent (Fig. 1). Others have reported that acquisition of rifampicin resistance can negatively impact growth rates although this probably depends on the exact mutation that is acquired in *rpoB* [22–24]. When grown as a colony on agar plates, the CR colonies had smooth edges and the CR strain lacked gliding motility, both changes that could be explained by either altered function of a mutated *rpoB* or other SNPs in relevant genes. For example, colonies of the CR grown on TYES and GMA media have reduced yellow pigmentation compared to the CW and CN strains. This phenotype may be attributed to the Thr365Lys mutation in the phytoene desaturase (*crtI*) gene, which encodes an enzyme involved in carotenoid biosynthetic pathway in *Flavobacterium* sp. [25–27]. Microscopic analysis of gliding motility and colony morphologies of TW, TN and TR strains did not reveal any phenotypic changes, despite the large number of SNPs that were accumulated by the TN and TR strains.

Polyacrylamide gel electrophoresis of LPS fractions revealed no obvious differences in LPS profiles, including no apparent changes from an Ile376Leu substitution in O-antigen acetylase (FPSM_01556) in the TR strain. 2D-PAGE analyses of proteins revealed probable differences in protein synthesis among rifampicin resistant *F. psychrophilum* strains (B17, CR and TR), which is consistent with our previous studies [14]. It is important to emphasize that while *rpoB* mutations might alter transcriptional regulation, other mutations in these strains could have contributed to this effect. Specifically, SNPs in proteins directly involved in gene/protein expression such as DNA primase, ribosome recycling factor, elongation factor P (EF-P), ATP-dependent RNA helicase (FPSM_01657) in the B17 or transcriptional regulator FPSM_00453 and ATP-dependent RNA helicase DbpA (FPSM_01345) in the TR strain.

Remarkably, considerable reduction in virulence of the CN (28.1 % mortality) and CR (4 % mortality) strains occurred despite relatively few SNPs leading to nonsynonymous amino acid changes (5 and 8, respectively). There were overlapping synonymous mutations among these strains and synonymous SNPs have been reported to potentially affect protein function [28]. Aside from SNPs occurring in the *rpoB* sequence, there was no overlap in SNPs for the attenuated B17 strain and the CR and CN strains developed in the current project. Noteworthy, 3 genes (*nrdA* – encoding a large chain of ribonucleotide-diphosphate reductase, *yihA* – encoding a GTP-binding protein and a putative membrane spanning protein FPSM_02361) out of 4 that harbor SNPs in the CN strain are identical with the CR strain. We surmise that the probability of these identical SNPs arising independently is unlikely. Instead, they most likely arose from the original culture used to initialize the passage experiments (i.e., a founder effect), or a cross-over contamination event occurred sometime early in the course of the experiment; if this was a contamination event, it must have occurred early in the experiment because these strains have distinct *rpoB* mutations making it easy to differentiate the final cultures. One of these shared SNPs is in *nrdA*, which has been implicated in pathogenesis of *Pseudomonas aeruginosa* [29]. Additionally, in *Escherichia coli yihA* is a GTPase that is essential for normal cell morphology and coordination of division; thus SNPs in this gene may contribute to attenuation [30, 31]. Because adhesion is an essential process in host-pathogen interaction and pathogenesis, mutation in the putative membrane spanning protein FPSM_02361 may have a direct effect on reduced virulence of CN and CR strains. Collectively, these findings are consistent with involvement of mutational events in the process of attenuation.

The CR strain was mostly attenuated (4 % mortality) and was characterized by slower growth in culture, lost gliding motility, three observable changes in protein synthesis, and altered carotenoid metabolism that is probably due to mutated phytoene desaturase (*ctrI*). As part of a gene cluster involved in carotenoid biosynthesis, mutations in *ctrI* were shown to alter colony pigmentation of other pathogens [32]. Importantly, mutations of *ctr* genes are able to decrease resistance to oxygen radicals and reduce growth in macrophages of the fish pathogen, *Mycobacterium marinum* [33]. Additionally, golden pigment synthesized through carotenoid pathway in *Staphylococcus aureus* enhances virulence by promoting resistance to respiratory burst of neutrophils, and the ∆*crtM* strain of *S. aureus* was also attenuated in mice model [34].

Additional SNPs unique to the attenuated CR strain included those found in a hypothetical protein (FPSM_01200), a transcriptional regulator (TetR family protein, FPSM_02533) and the Ser492Phe substitution in *rpoB*. The FPSM_01200 hypothetical protein has a predicted function of an N-acetylglucosamine (NAG) kinase, an enzyme important for bacterial cell wall and LPS metabolism. Moreover, importance of NAG metabolism was implied in initiation of murine intestine colonization by *E. coli* [35] and in bacterial signaling and growth in mucus of *P. aeruginosa* [36]. Together, these missense mutations may affect the virulence of the CR strain and its ability to colonize host and survive in challenged fish. In our analysis, however, we cannot discount the possible effects of silent SNPs on phenotype changes as described by Kimchi-Safraty and co-workers [28].

Our alternative hypothesis that *rpoB* can be directly involved in attenuation is potentially supported by the fact that RpoB is crucial in bacterial transcription and allosteric changes of the mutant protein may produce global alterations such as phenotype changes. The potential involvement of *rpoB* Ser492Phe mutation in attenuation of the CR strain is supported by existence of phenotypic (e.g. loss of gliding motility) and proteomic changes, and by a Gln474Arg *rpoB* mutation in the completely attenuated B17 strain [13, 14]. Furthermore, in other bacteria different *rpoB* mutations lead to different phenotypic changes [22–24, 37–39]. In *Brucella* spp. virulence and colony roughness of Rif^R^ phenotypes also varied depending on the position and character of single amino acid substitutions of RpoB [17]. Mutations providing resistance to rifampicin are clearly not universally responsible for attenuation because presumptively virulent Rif^R^ strains of *Mycobacterium tuberculosis* have been described [40–42].

Unlike attenuated strains derived from CSF 259–93, the TN and TR strains from wild-type THC 02–90 accumulated a large number of SNPs from serial passage regardless of the presence of rifampicin, and yet these changes had no apparent effect on virulence. The large numbers of SNPs present in the TN and TR strains as compared to the CN and CR strains may be attributed to the fact that the CSF 259–93 and THC 02–90 belong to different genetic lineages of *F. psychrophilum*. These two lineages are generally associated with different fish hosts, and the lineage to which THC 02–90 belongs is amenable to some genetic manipulations, which have not been successful with the lineage of strains to which CSF 259–93 belongs [19, 43–46]. It is notable that 54 and 46 % of the nonsynonymous mutations found in the TN and TR strains, respectively, occurred in subtilisin-like proteases that are putative cell surface proteins with predicted leucine-rich repeats. Repeat regions in DNA sequences are notoriously difficult to assemble correctly; particularly when using short read technologies such as employed herein [47, 48]. Notably, however, these same regions are also found in the CSF 259–93 genome and yet there were no mutations found in these gene sequences for the CN and CR strains. Given that all of these passaged strains introduced in this study were developed and sequenced at the same time using the same chemistries, we submit that this is not a sequencing artifact but that there is probably a distinctly different mutation process underway between the CSF 259–93 strains and the THC 02–90 strains. More strains from the two lineages need to be tested to determine if this is a lineage-level difference or if THC 02–90 is unique in this regard.

Additional changes at the SNP level, such as a SNP in *nrdA* in CR, and mutation in the FPSM_00026 gene encoding multimodular transpeptidase-transglycosylase PBP 1A and *mreC* in the B17 also support a mutation-dependent attenuation hypothesis because these genes have been associated with virulence (e.g. *nrdA* in *P. aeruginosa* [29], PBP 1A in group B streptococci [49], and MreC in *Salmonella* [50]). Alternatively, if *rpoB* mutation is more important, then the exact position of the mutations may be important. For example, TR was not attenuated, but it also had distinctly different mutations in the *rpoB* (Asp477Tyr and Pro496Ser) as compared to single amino acid substitutions of the mostly attenuated CR (Ser492Phe) and the fully attenuated B17 (Gln474Arg) strains. The large differential in SNP accumulation between passaged strains of CSF 259–93 and THC 02–90 are consistent with differential mutation process and coincidently may reflect differences at the lineage level [19].

Unfortunately, there are no molecular tools allowing for universal allelic exchange of genes in *F. psychrophilum* [44, 51]. Therefore, there is no direct way of assessing *rpoB* involvement in the process of rifampicin-induced attenuation. Introduction of the mutant *rpoB* allele from the B17, CR and TR into the wild-type *F. psychrophilum* would be the most direct method to investigate the role of rifampicin-resistant RNA polymerase in attenuation of these bacterial strains. Other options to characterize of attenuation in the CN, CR and B17 strains would be comparison of transcriptomes or more in-depth analysis of DNA methylation patterns between these and the virulent CSF 259–93 strain to examine possible global transcriptional changes from *rpoB* mutations or altered DNA methylation that might contribute to differential transcriptional regulation.

Differential DNA methylation may be associated with loss of virulence as altered DNA methylation affects these traits in other bacterial species [52, 53]. Others have found evidence for different methylation patterns among *F. psychrophilum* strains using restriction enzyme analysis [44, 54, 55]. Consequently, it was not entirely unexpected to find differences in methylation motifs based on the PacBio analysis (Additional file 1: Tables S3 and S4). It is remarkable, however, that there were no shared motifs between these two strains. Unfortunately, this also means that the comparison of the CR and TR strains provides no insight into the potential contribution of methylation to attenuation. Nevertheless, given that methylation and restriction enzymes can function to defend bacteria from foreign DNA, it is tempting to speculate that the two lineages of *F. psychrophilum* have diverged due to the influence of different phage communities. CSF 259–93 is most closely associated with freshwater fisheries whereas the THC 02–90 lineage is most closely associated with anadromous fish. It is likely that phage communities differ substantially between these ecosystems.

## Conclusions

Our findings demonstrate that the two *F. psychrophilum* strains passaged with rifampicin harbored from 27.6 % (for THC 02–90) to 33.33 % (for CSF 259–93) more SNPs than the paired strains that were passaged without the antibiotic. Importantly, passaged THC 02–90 strains, regardless of rifampicin presence in media, revealed considerably more SNPs (roughly 20-fold) than the CSF 259–93 strains, and that difference is correlated with the fact that these two strains belong to two genetically divergent lineages [19]. We also present data consistent with distinctly different methylation motifs between these two lineages. We observed almost complete attenuation of CSF 259–93 passaged with rifampicin (CR) and significant loss of virulence of the CSF 259–93 strain passaged without antibiotic (CN). These reductions of virulence of the CN (28.1 % mortality) and CR (4 % mortality) strains occurred despite a limited number of SNPs, 6 and 9, respectively, as compared to 31 SNPs of the fully attenuated B17 strain. The B17 strain shares no SNPs with the CN or CR strains, although both B17 and CR share a loss of gliding motility. There are several additional SNP’s that could collectively contribute to reduced virulence.

## Methods

### Generation of rifampicin resistant F. psychrophilum strains

*F. psychrophilum* CSF-259-93 [19] and THC 02–90 [56] were used as parent strains to generate rifampicin resistant strains. Previously frozen glycerol stocks of *F. psychrophilum* CSF-259-93 (hereafter referred to as CW) and THC 02–90 (hereafter referred to as TW) were plated for isolation on tryptone yeast extract salts (TYES; 0.4 % tryptone, 0.04 % yeast extract, 0.05 % MgSO_4_ × 7 H_2_O, 0.05 % CaCl_2_ × 2 H_2_O, pH 7.2) agar and incubated at 16 °C for 5 days [57]. Subsequently, single colonies were passed to TYES agar containing 10 μg/ml of rifampicin (Sigma, St. Louis, MO, USA) and incubated at 15 °C for 6 days. Two of the resulting colonies were then selected, designated CSF 259–93. AR and THC 02–90. AR, and independently passed to TYES agar containing increasing concentrations of rifampicin (initially every 10 μg/ml per passage and 25 μg/ml after 100 μg/ml). This process was repeated until the CSF 259–93. AR and THC 02–90. AR strains achieved growth at Rif concentrations of 250 μg/ml. This required 17 passages with 259-93AR strain designated as 259–93. AR17 (hereafter designated as CR) and 17 passages for the THC 02–90. AR17 (hereafter designated as TR) strain. Following each passage, a portion of the recovered cells was harvested, resuspended in sterile 100 % glycerol and frozen at −80 °C. The rifampicin resistant CSF 259-03B.17 (hereafter designated as B17 strain) used in our experiments was independently isolated by LaFrentz and coworkers [13].

### Passaging of F. psychrophilum strains without rifampicin

Previously frozen virulent CSF 259–93 and THC 02–90 strains were plated for isolation on TYES agar and incubated at 16 °C for 5 days. Two of the resultant colonies were selected for subsequent passages on TYES agar (total of 17 passages) leading to generation of CSF 259–93. N17 (hereafter referred to as CN) and THC 02–90. N17 strains (hereafter referred to as TN). Following each passage, a portion of the recovered cells was harvested, resuspended in sterile 100 % glycerol and frozen at −80 °C.

### Growth comparison and bacterial cultures

A Bioscreen C system and EZ Experiment software (Growth Curves USA) were used to measure optical density of cultures to examine possible differences for in vitro growth kinetics. Briefly, bacterial strains were cultured statically in 5 ml of TYES broth from previously frozen stocks. After 3 days of incubation at 16 °C the cultures were diluted with TYES broth to OD 0.3 (at 600 nm) and loaded onto the Bioscreen C system. We used 10 × 10 honeycomb microplates, with triplicates of every strain (in 200 μl TYES broth per well) being statically incubated for 5 days at 16 °C with 10 s shaking before each measurement. The optical density measurements were collected using a 450–580 nm bandwidth filter every 3 h.

*F. psychrophilum* strains (CW, CN, CR, TW, TN, TR and B17) used later for genomic DNA or protein extractions, and for fish challenge were grown statically in 25 ml of TYES broth at 16 °C for 3 days.

### Microscopy

Bacterial cell morphology was examined with Gram staining of heat-fixed bacteria obtained from TYES broth cultures that were incubated statically for 3 days at 16 °C. Colony morphology was examined with isolated colonies growing on TYES agar and gliding motility was observed by stab inoculation of gliding motility agar (GMA; 0.8 % nutrient broth and 0.75 % agar), similar to procedures described by Perez-Pascual and co-workers [58]. Both TYES and GMA plates were incubated for 3 days at 16 °C. Bacterial colonies were observed under 200× magnification with a Leica microscope equipped with an EC-3 digital camera. Leica Application Suite EZ (LAS EZ) software was used for image acquisition and analysis (Leica Microsystems). At least three independent biological and technical replicates per media type per strain were used.

### rpoB and 16S rRNA analysis

To ensure culture purity before whole-genome sequencing, *rpoB* sequencing and 16S rRNA analysis were performed. Briefly, genomic DNA was isolated from 5 ml TYES cultures of the parent and passaged strains of *F. psychrophilum* with QIAamp DNA Mini Kit (QIAgen) according to the manufacturer’s instructions. External primers for *rpoB* amplification were 5′-AAAATCGGAACGGATTACGG-3′ and 5′-TTTTGAATTGTTTTTAAAGAGGTATTG-3′, and PCR involved 35 cycles of 94 °C for 30 s, 45 °C for 30 s and 68 °C for 4 min. Primers used to amplify internal *rpoB* segments for sequencing were described previously by Gliniewicz and coworkers [14]. All reactions included 2 mM MgSO_4_, 1 × PCR buffer, 0.2 mM dNTP mixture, 1 U of HiFi *Taq* polymerase (Invitrogen) with 5 ng DNA template and 0.1 μM of each primer in 50 μl reaction volume. 16S rRNA analysis was performed according to method described by Soule and coworkers with 16S_336fwd and 16S_517rvs primers used for target amplification and *MaeIII* digestion of amplified 16S rRNA to distinguish CSF 259–93 and THC 02–90 strains [19].

### One-dimensional SDS-PAGE

Protein electrophoresis followed Laemmli (1970) with some modifications. Prior to electrophoresis 2 ml cultures of parent and passaged strains of *F. psychrophilum* (OD_595_ 0.6) were centrifuged, cells were resuspended in 0.5 ml PBS and diluted 1:2 in sample buffer containing a reducing agent (100 mM β-mercaptoethanol) and boiled for 5 min. Proteins from bacterial whole-cell lysate were separated using pre-cast Any-kD polyacrylamide gels (Bio-Rad). Gels were used in a Mini-PROTEAN 3 electrophoresis cell (Bio-Rad) at 120 V for 70 min. Proteins were stained with Coomassie Blue and Precision Plus protein standards (Bio-Rad) were used to estimate the molecular mass of proteins. Analysis of carbohydrate extractions was conducted according to the method of LaFrentz and co-workers [13] and LPS fractions were silver stained with Pierce Silver Stain Kit according to manufacturer’s instruction (Thermo Scientific, USA). ChemiDoc XRS system and Image Lab 4.1 software were used for visualization (Bio-Rad).

### Two-dimensional PAGE

Two-dimensional PAGE (2D PAGE) of *F. psychrophilum* proteins was performed as described earlier by Gliniewicz and co-workers [14]. Briefly, proteins from whole-cell lysates of *F. psychrophilum* strains were subjected to alkylation reduction and cleaned with 2D clean-up kits according to the manufacturer’s instructions (BioRad). Protein concentrations were estimated by using a QuickStart Bradford kit (BioRad). Samples from each strain with ~250 μg/μl protein per strip, were applied to immobilized pH gradient (IPG) strips (11 cm, pH 3–10) by passive rehydration for 1 h. Rehydrated IPG strips were covered with mineral oil and incubated overnight at room temperature. First dimension isoelectric focusing (IEF) was performed using a Protean IEF Cell (BioRad) with 0–250 V for 15 min, 0–8000 V rapid ramp for 40,000 Vh with 50 μA per strip. IPG strips were subsequently treated with DTT (2 % w/v) and iodoacetamide (2.5 % w/v) dissolved in equilibration buffer (6 M urea, 0.357 M Tris–HCl, pH 8.8, 2 % SDS, 20 % glycerol) with gentle rocking for 10 min. Second dimension separation was accomplished by application of IPG strips onto polyacrylamide gels with a linear 10–20 % Tris–HCl gradient and electrophoresis with Criterion Gel System (BioRad) and resolved at 200 V for 55 min. Gels were stained with SyPro Ruby Protein Stain according to the manufacturer’s directions. Digital images were collected using a Fluor-S Multi Imager (BioRad) and Quantity One software was used for comparison of protein profiles between bacterial strains (BioRad). For our discussion we only noted differences in protein profiles that were consistent across three biological replicates each having three technical replicates.

### Genomic DNA extraction

Genomic DNA of the parent and passaged strains of *F. psychrophilum* were isolated with QIAamp DNA Mini Kit according to the manufacturer’s instructions (QIAgen) from 20 ml TYES broth cultures incubated at 16 °C for 5 days. DNA quality was analyzed with NanoDrop ND-1000 spectrophotometer (Thermo Scientific) and by agarose gel electrophoresis and visualization with ethidium bromide.

### High throughput DNA sequencing

Genomic DNA libraries for CW and CSF 259-93B.17 strains were bar-coded by ligating adapters incorporating molecular identifiers during robust long-library construction. The libraries were quantified by fluorescence on a VictorX 384 well plate fluorometer (Parkin-Elmer). Quantified libraries were titrated to yield 8 % labeling of DNA capture beads using small volume emulsion PCR (emPCR) reactions and enough beads for sequencing were obtained by pooling the titration beads with a single medium volume emPCR reaction. Beads (1 × 10^6^) for each strain were combined and used to load half of one 70 × 75 titanium picotiter plate. Sequencing was performed on a Roche 454 FLX Titanium instrument. Later, genomic DNA of six strains of *F. psychrophilum* (CW, CN, CR, TW, TN and TR) was subject to whole-genome sequencing with IonTorrent technology. Briefly, genomic DNA samples were fragmented using a Bioruptor 300 (Diagenode) for 37 min using a 30 s on-off cycling and temperature controlled sonication bath and had a peak apparent molecular weight of 300 nucleotide base pairs. The fragmented DNA was size selected and purified using a Pippin Prep elecro-elution device (Sage Science) set for a tight range harvest at 315 bp. Library construction was performed using the Ion Fragment Plus library kit (Life technologies). Library quantification and amplification efficiency was determined by real-time PCR. An appropriate number of library molecules were used to achieve 20 % bead labeling. Emulsion PCR and bead harvesting was performed on Ion One-Touch/One Touch ES instruments (Life technologies). Percent DNA bead labeling and number of beads harvested was determined using Sybr Gold stained beads and a guava easyCyte flow cytometer (Millipore). Sequencing was performed on an Ion Torrent PGM running Torrent Suite software version 2.0.1 (Life technologies). All strains were sequenced separately using a single 316 chip for each strain. Sequencing experiments were conducted in the Molecular Biology and Genomics Core at Washington State University. This whole genome shotgun project is available at DDBJ/EMBL/GenBank under the accession JRWA00000000, JRWB00000000, JRWC00000000, JRWD00000000, JRWE00000000, JRWF00000000. The version described in this paper is version JRWX01000000 where X is A, B, C, D, E, or F.

### Single-nucleotide polymorphism analysis

CLC Genomic Workbench 5.0 (CLC bio) with default parameters was used for contig assembly, SNP detection and analysis of amino acid substitutions. For SNP detection, The Quality Based Variant Detection toolbox in CLC was used, with the following minor modifications to default settings. A minimum coverage of 4 was required with a variant frequency of 80 %. Only variants that resulted in an amino acid substitution in a coding sequence are reported. *F. psychrophilum* CSF 259–93 genome of 2,900,735 bp was used as reference (GenBank Accession number: CP007627) [59]. For the analysis of TN and TR strains the SNPs were compared with the sequenced parent TW and CSF 259–93 parent strains, and the *F. psychrophilum* JIP 02/86 genome (NCBI Reference Sequence: NC_009613.3).

### DNA methylation analysis

Two *F. psychrophilum* strains (CR and TR) were selected for analysis of possible changes of DNA methylation patterns. Large-fragment libraries were created from 5 μg of genomic DNA from each strain. DNA was sheared at 20× speed code 15 through a large aperture ruby of a DigiLab Hydroshear Plus device (DigiLab, USA) and a larger-fragment library was constructed using the PacBio DNA Template Kit for 3–10 kb fragments (Pacific Biosciences, USA). Short-fragment libraries were constructed from 5 μg of genomic DNA from each strain with the DNA sheared at 20× speed code 6 through a small aperture ruby of a DigiLab Hydroshear Plus device (DigiLab, USA) device and prepared using the PacBio DNA Template Kit for 250 bp – 3 kb fragments (Pacific Biosciences, USA). The resulting libraries were quantified using a Qbit fluorometer (Life Technologies, USA) and size range determined on an Agilent 2100 Bioanlayzer using the DNA12000 kit (Agilent Technologies, USA). Peak size distributions of libraries were approximately 10 kb and 2 kb for the large and short library, respectively. The large libraries were annealed to primer, bound to C2 polymerase, loaded with magnetic beads and observed on 5 SMRT cells per library using single 120 min movies (Pacific Biosciences, USA). The short libraries were annealed to primer, bound to C2 polymerase, diffusion loaded into 4 SMRT cells per library, and observed using two 45 min movies per cell. Cumulatively, 13 movies from 9 SMRT cells were collected for each strain. Allora and HGAP software were used for assembly and analysis of the data (Pacific Biosciences, USA).

### Fish rearing conditions

Rainbow trout (mean weight 1 g) were used to assess virulence of six *F. psychrophilum* strains. Fish were stocked in 19-liter flow-through tanks (25 fish per tank) supplied with dechlorinated municipal and fed 1.4 mm pelleted trout food (Rangen, Inc.) at 1 % body weight per day. Water temperature was maintained at 14 °C throughout the challenge experiments. The fish had no previous history of *F. psychrophilum* infection.

### Assessment of attenuation

Fish were challenged by intraperitoneal injection with a 30-gauge needle with 25 μl of *F. psychrophilum* culture (OD_595_ = 0.2) resuspended in 25 μl of PBS. Triplicate groups of 25 fish were challenged with each *F. psychrophilum* strain or with PBS as a mock infected control group of 25 fish. An OD of 0.2 correlated to ~1.5 × 10^8^ colony forming units (cfu)/ml and was estimated using the 6 × 6 drop plate method [60]. Mortalities were recorded daily for 28 days and kidney, liver and spleen tissues were streaked onto TYES agar to confirm the presence of yellow-pigmented bacteria that were presumptive *F. psychrophilum*. The University of Idaho Institute for Animal Care and Use Committee approved all animal husbandry and experimental challenge procedures.

### Statistical analysis

SigmaPlot version 12 (Systat Software, Inc.) was used to calculate the areas-under-the-curve (AUC) for in vitro growth curves. NCSS ver. 7.1.19 software (NCSS, LCC) was used for one-way ANOVA with Tukey’s *post hoc* tests of endpoint and AUC data. Log-rank Mantel-Cox test was used to compare fish survival rates among different treatments. GraphPad Prism software (GraphPad Software, Inc.) was used for statistical analyses and for graph preparation.

## Additional file

Additional file 1: Figure S1. SDS-PAGE analysis of carbohydrate extractions from different *F. psychrophilum* CSF 259-93 and THC 02-90 strains. **Table S1**: Single nucleotide polymorphisms (SNPs) from *F. psychrophilum* THC 02-90 strain passaged without rifampicin (TN). **Table S2**: Single nucleotide polymorphisms (SNPs) from *F. psychrophilum* THC 02-90 strain passaged with rifampicin (TR). **Table S3**: DNA methylation motifs unique for the rifampicin passaged CSF 259-93 strain (CR). **Table S4**: DNA methylation motifs unique for the rifampicin passaged THC 02-90 strain (TR). (DOCX 637 kb)
