# Supplementary material for: Potential mechanisms of attenuation for rifampicin-passaged strains of Flavobacterium psychrophilum
Source: BMC Microbiol. 2015 Sep 16;15:179. doi: 10.1186/s12866-015-0518-1 (PMC4571129; doi:10.1186/s12866-015-0518-1)
Supplement: Additional file 1: Figure S1. — SDS-PAGE analysis of carbohydrate extractions from different F. psychrophilum CSF 259-93 and THC 02-90 strains. Table S1: Single nucleotide polymorphisms (SNPs) from F. psychrophilum THC 02-90 strain passaged without rifampicin (TN). Table S2: Single nucleotide polymorphisms (SNPs) from F. psychrophilum THC 02-90 strain passaged with rifampicin (TR). Table S3: DNA methylation motifs unique for the rifampicin passaged CSF 259-93 strain (CR). Table S4: DNA methylation motifs unique for the rifampicin passaged THC 02-90 strain (TR). (DOCX 637 kb) [file 12866_2015_518_MOESM1_ESM.docx]

**Supplemental data**

**Potential mechanisms of attenuation for rifampicin-passaged strains of *Flavobacterium* *psychrophilum***

Karol Gliniewicz^1^, Mark Wildung^2^, Lisa H. Orfe^7^, Gregory D. Wiens^3^, Kenneth D. Cain^4^, Kevin K. Lahmers^5^, Kevin R. Snekvik^1,7^ and Douglas R. Call^1,7*^

Fig. S1. SDS-PAGE analysis of carbohydrate extractions from different *F. psychrophilum* CSF 259-93 and THC 02-90 strains.

Table S1. Single nucleotide polymorphisms (SNPs) from *F. psychrophilum* THC 02-90 strain passaged without rifampicin (TN)

Table S2. Single nucleotide polymorphisms (SNPs) from *F. psychrophilum* THC 02-90 strain passaged with rifampicin (TR).

Table S3. DNA methylation motifs unique for the rifampicin passaged CSF 259-93 strain (CR).

Table S4. DNA methylation motifs unique for the rifampicin passaged THC 02-90 strain (TR).

Fig. S1. SDS-PAGE analysis of carbohydrate extractions from different *F. psychrophilum* CSF 259-93 and THC 02-90 strains. Gel was silver stained and molecular weight markers (M) are indicated on the left of the gel. *Salmonella* Typhimurium LT-2 strain was used as a control (C). CW – wild-type CSF 259.93; CN – CSF 259-93 passaged without rifampicin; CR– CSF 259-93 passaged with rifampicin; B17 – attenuated CSF 259.93B.17; TW – wild-type THC 02-90; TN – THC 02-90 passaged without rifampicin; TR – THC 02-90 passaged with rifampicin.


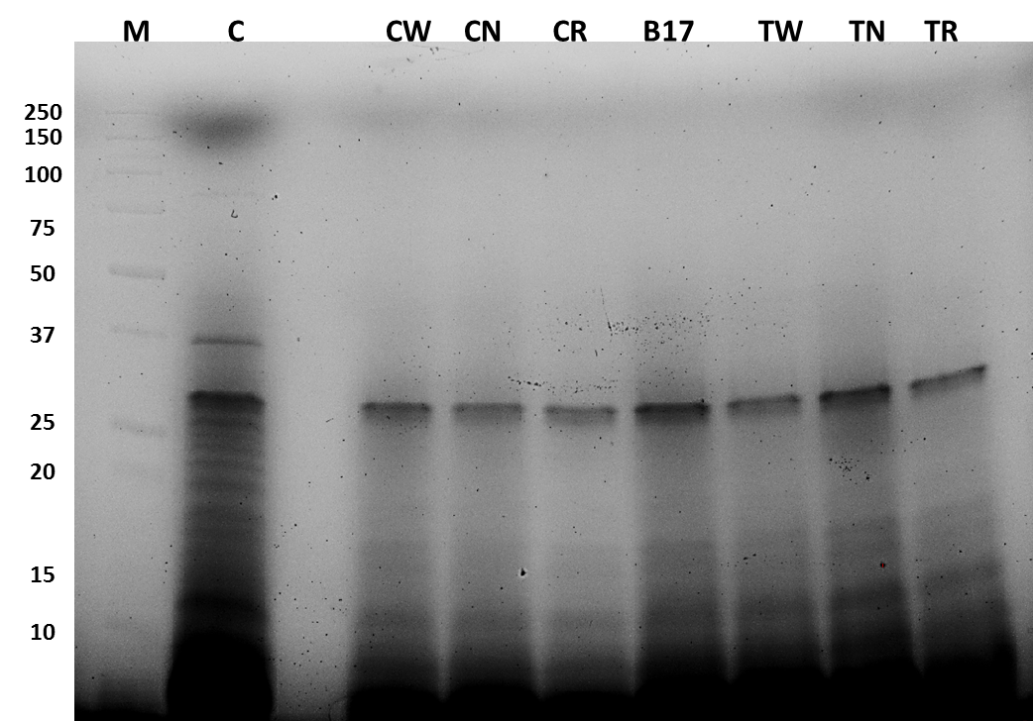


Table S1. Single nucleotide polymorphisms (SNPs) from *F. psychrophilum* THC 02-90 strain passaged without rifampicin (TN).

| **Reference position^a^** | **Allele frequency (in %)^b^** | **SNP^c^** | **Coverage^d^** | **Annotation^e^** | **Amino acid change^e^** | **Gene / Protein^f^** |
| --- | --- | --- | --- | --- | --- | --- |
| 27440 | 100 | C > A | 10 | FPSM_00017 | His1247Asn | PKD domain protein |
| 216039  216045  216297  216298  216307  216322 | 100  100  90  95  90  81.5 | T > C  C > T  T > G  T > A  C > G  C > T | 10  9  20  20  30  27 | FPSM_00182 | Glu162Gly  Gly160Glu  Asn76Thr*  Asn76Tyr*  Glu73Gln  Gly68Arg | Subtilisin-like protease |
| 216657 | 86.2 | G > A | 29 | FPSM_00183 | Thr17Ile | Hypothetical protein |
| 217136  217497  217556  217721  217722 | 88.5  100  100  85.4  88.6 | C > G  C > A  T > G  G > T  T > C | 26  4  7  41  41 | FPSM_00184 | Gly213Ala  Ala93Ser  Glu73Ala  Thr18Asn*  Thr18Ala* | Subtilisin-like protease |
| 218661  218665  218803  218860 | 81.2  88  85.7  90 | T > C  C > T  C > T  C > T | 16  25  21  10 | FPSM_00185 | Glu278Gly  Asp277Asn  Asp231Asn  Ala212Thr | Subtilisin-like protease |
| 220503  220510  220639 | 100  83.3  86.4 | C > T  C > A  G > C | 4  6  22 | FPSM_00186 | Gly68Glu  Ala66Ser  His23Asp | Subtilisin-like protease |
| 222096  222097  222098  222234  222315  222438  222442  222622 | 86.3  82.1  86.4  100  100  100  100  100 | T > G  C > A  C > A  T > C  T > G  A > G  A > T  T > C | 51  28  22  7  6  6  6  4 | FPSM_00188 | Asp208Ala*  Asp208Tyr*  Glu207Asp  Glu162Gly  Asp135Ala  Val94Ala  Tyr93Asn  Thr33Ala | Subtilisin-like protease |
| 223461  223495  223576 | 100  100  81.4 | A > G  C > T  C > G | 16  7  59 | FPSM_00189 | Ile111Thr  Gly100Ser  Val73Leu | Subtilisin-like protease |
| 224452  224779 | 100  93.9 | C > A  G > A | 4  31 | FPSM_00190 | Ala116Ser  Leu7Phe | Subtilisin-like protease |
| 224896  225309  225310  225370  225394  225462  225468  225708  225709  225728  225736 | 92.9  83.3  83.3  83.3  88.9  100  100  100  100  100  83.3 | C > T  A > T  A > C  A > T  T > C  T > C  T > G  T > G  C > T  T > A  G > C | 14  6  6  12  9  4  4  7  5  5  6 | FPSM_00191 | Val303Ile  Phe165Tyr*  Phe165Val*  Ser145Thr  Arg137Gly  Glu114Gly  Asp112Ala  Glu32Ala*  Glu32Lys*  Arg25Ser  His23Asp | Subtilisin-like protease |
| 226612  226650  226668  226669  226674  226675  226681  226699  226734 | 100  100  100  100  100  100  100  100  100 | T > C  T > G  G > C  A > G  C > T  C > T  C > A  T > C  A > G | 5  4  7  7  6  5  6  7  5 | FPSM_00192 | Thr89Ala  Asn76Thr  Ser70Cys*  Ser70Pro*  Gly68Glu*  Gly68Arg*  Ala66Ser  Lys60Glu  Val48Ala | Subtilisin-like protease |
| 227116  227695  227787  227788  227814 | 88.9  100  81.8  85.7  90.9 | C > T  C > T  A > G  C >T  C > T | 9  6  11  7  22 | FPSM_00193 | Glu256Lys  Ala63Thr  Val32Ala*  Val32Ile*  Gly23Asp | Subtilisin-like protease |
| 228592  228593  228603  228987  229262  229275  229331 | 89.5  89.5  81  81.2  81.2  84.6  100 | G > C  C > A  G > A  A > C  A > G  C > T  C > T | 19  19  21  48  16  13  6 | FPSM_00194 | Ser271Arg*  Ser217Ile*  His268Tyr  Ser140Ala  Val48Ala  Asp44Asn  Arg25Lys | Subtilisin-like protease |
| 230009  230258  230408 | 100  90.5  83.3 | A > G  T > C  T > G | 4  21  6 | FPSM_00195 | Ile226Thr  Asn143Ser  Tyr93Ser | Subtilisin-like protease |
| 235056  235387 | 81.4  100 | C > G  A > T | 59  4 | FPSM_00200 | Gly291Ala  Ser181Thr | Subtilisin-like protease |
| 305600  305795  305889 | 81.8  96.6  100 | T > C  A > T  T > A | 33  29  5 | FPSM_00269 | Thr129Ala  Phe64Ile  Lys32Asn | Hypothetical protein |
| 306282  306326 | 100  100 | T > A  G > T | 11  9 | FPSM_00270 | Lys52Asn  Leu38Ile | Hypothetical protein |
| 306672 | 100 | C > T | 17 | FPSM_00271 | Ser7Asn | Hypothetical protein |
| 399622  399670  399676 | 83.3  90.5  90 | T > C  G > T  G > T | 12  21  20 | FPSM_00355 | Tyr186His  Ala202Ser  Asp204Tyr | Endoglucanase |
| 521257  521326 | 87.5  100 | G > A  T > C | 24  9 | FPSM_00453 | Thr120Ile  Glu97Gly | Transcriptional regulator |
| 615725 | 100 | A > C | 4 | FPSM_00537 | Ile175Met | Transposase |
| 647100 | 100 | A > T | 6 | FPSM_00567 | Arg168Gln | Hypothetical protein |
| 661078  662593 | 100  100 | A > T  C > A | 9  8 | FPSM_00574 | Asn599Lys  Lys94Asn | Virulence-associated protein E |
| 664918  664926  664927  664944  664945  664141 | 100  100  100  100  100  100 | A > T  T > G  T > A  T > G  T > C  T > G | 19  16  15  17  12  4 | FPSM_00578 | Phe304Tyr  Gln301His*  Gln301Leu*  Lys295Asn  Lys295Ile  Lys230Glu | Integrase/recombinase (XerC/CodV family) |
| 675603 | 100 | C > T | 4 | FPSM_00587 | Ser56Phe | Transposase |
| 798861 | 85.3 | T > G | 34 | FPSM_00695 | Lys189Gln | Hypothetical protein |
| 989475 | 82.1 | T > G | 84 | FPSM_00879 | Asn444Lys | Outer membrane porin F |
| 1184740 | 81.8 | T > A | 33 | FPSM_01063 | Ile28Asn | Hypothetical protein |
| 1205520 | 100 | A > T | 5 | FPSM_01080 | Phe37Tyr | 3-ketoacyl-CoA thiolase |
| 1368772  1368784  1368800  1368806  1368847  1368973  1368988 | 100  100  100  100  100  83.3  100 | G > C  A > C  A > G  T > A  C > T  T > A  T > C | 8  6  7  9  5  12  11 | FPSM_01243 | Glu12Gln  Ile16Leu  Lys21Arg  Val23Asp  His37Tyr  Leu79Ile  Tyr84His | ATP/GTP-binding protein |
| 1392255  1382271  1392292 | 100  100  100 | C > G  C > T  C > T | 5  5  4 | FPSM_01261 | Trp44Cys  Ser39Asn  Arg32Lys | Hypothetical protein |
| 1404932  1405000  1405001  1405012  1405023 | 100  100  100  100  100 | A > T  C > G  A > C  A > C  G > A | 8  7  7  7  9 | FPSM_01279 | Asn236Ile  Gln259Glu*  Gln259Pro*  Lys263Gln  Met266Ile | Hypothetical protein |
| 1405154  1405160  1405781 | 100  100  100 | A > T  C > T  C > A | 6  5  11 | FPSM_01280 | Ser43Cys  Pro45Ser  Leu252Ile | ATP/GTP-binding protein |
| 1454475 | 81.1 | A > T | 37 | FPSM_01312 | Ile7Phe | Hypothetical protein |
| 1964490 | 83.3 | T > A | 6 | FPSM_01781 | Phe166Ile | Transposase |
| 1980606 | 88.2 | T > C | 76 | FPSM_01797 | Val282Ala | Folylpolyglutamate synthase |
| 2344276 | 100 | G > T | 8 | FPSM_02112 | Val354Leu | Lysyl-tRNA synthetase |
| 2507678 | 100 | G > A | 6 | FPSM_02284 | Thr160Ile | Putative ATPase |
| 2508748 | 92.1 | A > G | 114 | FPSM_02286 | Ile279Thr | Hypothetical protein |
| 2550969 | 100 | A > C | 11 | FPSM_02315 | Asn267Thr | Putative membrane associated protein |
| 2593282 | 100 | A > T | 5 | FPSM_02351 | Leu364Phe | Multimodular transpeptidase-transglycosylase |
| 2697370 | 100 | C > A | 5 | FPSM_02440 | Pro25His | Hypothetical protein |
| 2744838 | 100 | T > G | 4 | FPSM_02492 | Asp943Glu | Leucyl-tRNA synthetase |
| 2748069 | 83.6 | C > A | 61 | FPSM_02497 | Gln212Lys | Acylamino-acid-releasing enzyme |
| 2806175 | 89.7 | A > T | 68 | FPSM_02542 | Ile7Asn | Putative membrane spanning protein |

^*^Denotes undetermined SNP call

^a^ Reference position (in bp) for reference sequence (listed under annotation field) are based on CSF 259-93 sequence from ERGO-Integrated genomics

^b^ Allele frequency refers to the proportion of sequences showing a given single-nucleotide polymorphism (SNP) at the started reference position

^c^ Shows the SNP change represents the change from nucleotide X to Y (X > Y) at the reference position

^d^ Coverage refers to the total number of sequencing reads that align to each base within the sample DNA

^e^ Predicted amino acid change for the identified SNP

^f^ Annotation shows the name and putative function for the identified gene. Annotation is presented from the CSF 259-93 sequence from ERGO-Integrated genomics Table S2. Single nucleotide polymorphisms (SNPs) from *F. psychrophilum* THC 02-90 strain passaged with rifampicin (TR).

| **Reference position^a^** | **Allele frequency (in %)^b^** | **SNP^c^** | **Coverage^d^** | **Annotation^e^** | **Amino acid change^e^** | **Gene / Protein^f^** |
| --- | --- | --- | --- | --- | --- | --- |
| 27071  27077  27084  27101  27108  27111  27117  27123  27132  27147  27155  27440 | 100  87.5  100  88.9  88.9  87.5  100  87.5  83.3  87.5  90.9  100 | G > C  G > A  T > C  A > C  A > T  A > C  G > A  A > C  T > C  C > A  A > G  C > A | 7  8  7  9  9  8  5  8  12  8  11  19 | FPSM_00017 | Ala1124Pro  Ala1126Thr  Leu1128Pro  Thr1134Pro  Tyr1136Phe  Asp1137Ala  Ser1139Asn  Asn1141Thr  Ile1144Thr  Thr1149Asn  Ser1152Gly  His1247Asn | PKD domain protein |
| 164919 | 96.6 | T > C | 89 | FPSM_00139 | Lys54Glu | Tetratricopeptide repeat family protein |
| 216297  216298  216307  216322  216328 | 96.7  89.3  91.8  84.8  81 | T > G  T > A  C > G  C > T  C > A | 30  28  49  46  63 | FPSM_00182 | Asn76Thr*  Asn76Tyr*  Glu73Gln  Gly68Arg  Ala66Ser | Subtilisin-like protease |
| 216999  217186  217502  217556  217567 | 83.6  83.3  100  83.3  100 | G > C  T > G  T > C  T > G  T > A | 61  6  4  6  4 | FPSM_00184 | Gln259Glu  Lys196Asn  Glu91Gly  Glu73Ala  Glu69Asp | Subtilisin-like protease |
| 218665  218860  218998 | 80.6  85.7  100 | C > T  C > T  A > T | 31  7  7 | FPSM_00185 | Asp277Asn  Ala212Thr  Ser166Thr | Subtilisin-like protease |
| 219551  219846  219876 | 87.5  100  85.7 | C > T  G > T  A > T | 8  25  28 | FPSM_00186 | Met385Ile  Thr287Lys  Val277Glu | Subtilisin-like protease |
| 222096  222097  222098  222100  222234  222271  222294  222304  222315  222316  222355  222364  222385  222387  222424  222499 | 88.7  81.1  86.7  81.8  100  82.6  90  100  100  81.8  100  100  88.9  100  84.6  83.3 | T > G  C > A  C > A  C > T  T > C  T > C  A > T  C > A  T > G  C > T  A > T  C > T  T > C  A > G  A > T  G > A | 71  37  30  33  9  23  10  4  12  11  6  5  9  10  13  12 | FPSM_00188 | Asp208Ala*  Asp208Tyr*  Glu207Asp*  Glu207Lys*  Glu162Gly  Ile150Val  Phe142Tyr  Gly139Cys  Asp135Ala*  Asp135Asn*  Ser122Thr  Gly119Ser  Thr112Ala  Ile111Thr  Ser99Thr  His74Tyr | Subtilisin-like protease |
| 222742  223461  223495  223522  223528  223576 | 100  95.5  100  81.8  84.6  95 | T > C  A > G  C > T  C > T  T > C  C > G | 7  22  9  66  91  100 | FPSM_00189 | Ile351Val  Ile111Thr  Gly100Ser  Gly91Arg  Thr89Ala  Val73Leu | Subtilisin-like protease |
| 224440  224452 | 90.9  100 | C > G  C > A | 11  10 | FPSM_00190 | Gly120Arg  Ala111Ser | Subtilisin-like protease |
| 225253  225300  225307  225309  225736 | 100  100  100  100  100 | T > C  A > G  C > G  A > T  G > C | 5  5  4  4  4 | FPSM_00191 | Lys184Glu  Phe168Ser  Gly166Arg  Phe165Tyr  His23Asp | Subtilisin-like protease |
| 226660  226669  226681  226699 | 100  100  100  100 | C > G  A > G  C > A  T > C | 4  4  4  4 | FPSM_00192 | Val73Leu  Ser70Pro  Ala66Ser  Lys60Glu | Subtilisin-like protease |
| 227116  227695  227787  227788  227814 | 88.9  100  81.8  85.7  90.9 | C > T  C > T  A > G  C > T  C > T | 9  6  11  7  22 | FPSM_00193 | Glu256Lys  Ala63Thr  Val32Ala*  Val32Ile*  Gly23Asp | Subtilisin-like protease |
| 228592  229311  229330  229331 | 81.2  87.5  83.3  85.7 | G > C  C > T  T > A  C > T | 16  8  6  7 | FPSM_00194 | Ser271Arg  Glu32Lys  Arg25Ser*  Arg25Lys* | Subtilisin-like protease |
| 230063  230064  230069  230075  230082  230111  230271  230468 | 100  100  100  100  100  92.3  100  88.5 | C > T  C > A  T > C  T > G  C > T  C > T  C > G  T > A | 4  4  4  4  8  13  9  104 | FPSM_00195 | Gly208Asp*  Gly208Cys*  Glu206Gly  Asp204Ala  Val202Met  Gly192Asp  Glu139Gln  Glu73Val | Subtilisin-like protease |
| 232429 | 80.4 | G > C | 163 | FPSM_00197 | Ser210Arg | Subtilisin-like protease |
| 233435  233436  233452  233459  233467 | 100  100  100  100  100 | G > A  T > C  A > T  A > C  C > G | 7  7  7  6  6 | FPSM_00198 | Thr163Ile*  Thr163Ala*  Phe157Leu  Ile155Arg  Gln152His | Subtilisin-like protease |
| 235387  235702  235711  235732  235761  235831 | 100  100  100  87.5  85.7  100 | A > T  C > T  C > G  C > A  G > A  T > C | 7  8  10  8  7  12 | FPSM_00200 | Ser181Thr  Asp76Asn  Glu73Gln  Ala66Ser  Ala56Val  Thr33Ala | Subtilisin-like protease |
| 306160  306282 | 100  100 | G > T  T > A | 17  11 | FPSM_00270 | Ala93Glu  Lys52Asn | Hypothetical protein |
| 306672 | 100 | C > T | 17 | FPSM_00271 | Ser7Asn | Hypothetical protein |
| 330534 | 100 | A > G | 6 | FPSM_00296 | Asp159Gly | Putative exported protein |
| 373366 | 90.5 | T > C | 84 | FPSM_00331 | Lys243Arg | Periplasmic component of efflux system |
| 399622  399670  399676  399737  401015 | 100  100  100  100  92.7 | T > C  G > T  G > T  A > T  T > C | 23  36  41  7  150 | FPSM_00355 | Tyr186His  Ala202Ser  Asp204Tyr  Thr291Ser  Val650Ala | Endoglucanase |
| 521257 | 89.2 | G > A | 37 | FPSM_00453 | Thr120Ile | Transcriptional regulator |
| 647100 | 100 | C > T | 5 | FPSM_00567 | Arg168Gln | Hypothetical protein |
| 661078  662593 | 100  100 | A > T  C > A | 10  7 | FPSM_00574 | Asn599Lys  Lys94Asn | Virulence-associated protein E |
| 664918  664926  664927  664944  664945 | 92.7  100  100  97  96.9 | A > T  T > G  T > A  T > G  T > A | 41  40  39  33  32 | FPSM_00578 | Phe304Tyr  Gln301His  Gln301Leu  Lys295Asn*  Lys295Ile* | Integrase/recombinase (XerC/CodV family) |
| 709541 | 100 | G > C | 4 | FPSM_00623 | His47Asp | Hypothetical protein |
| 798861 | 82.9 | T > G | 41 | FPSM_00695 | Lys189Gln | Hypothetical protein |
| 800969 | 81.9 | A > T | 116 | FPSM_00697 | His71Gln | Inosine-5’-monophosphate dehydrogenase |
| 989475 | 88.5 | T > G | 104 | FPSM_00879 | Asn444Lys | Outer membrane porin F |
| 990815 | 82 | G > A | 50 | FPSM_00880 | Thr425Ile | Tripeptidase T |
| 1017189 | 93.2 | A > G | 44 | FPSM_00904 | Ile218Thr | Putative peptidoglycan binding domain protein |
| 1116250 | 87.3 | G > C | 63 | FPSM_01000 | Asn115Lys | dATP pyrophosphohydrolase |
| 1296620  1296632  1296678  1296740 | 100  100  100  100 | A > G  G > A  G > T  A > G | 5  4  4  5 | FPSM_01170 | Lys370Glu  Val374Ile  Arg389Ile  Ile410Val | Mannosyltransferase |
| 1309880 | 91.2 | A > C | 34 | FPSM_01184 | Asn206Lys | Thiol:disulfide interchange protein DsbD |
| 1368522  1368560 | 100  100 | G > C  G > A | 4  4 | FPSM_01242 | Ala225Pro  Met237Ile | Hypothetical protein |
| 1368772  1368784  1368800  1368806  1368973  1368988 | 100  100  100  100  100  100 | G > C  A > C  A > G  T > A  T > A  T > C | 5  6  5  5  16  19 | FPSM_01243 | Glu12Gln  Ile16Leu  Lys21Arg  Val23Asp  Leu79Ile  Tyr84His | ATP/GTP-binding protein |
| 1392255  1392266  1382271  1392292  1392346  1392357  1392366 | 100  100  100  100  81.8  100  100 | C > G  C > A  C > T  C > T  C > T  T > G  T > G | 31  25  33  35  11  21  13 | FPSM_01261 | Trp44Cys  Gly41Cys  Ser39Asn  Arg32Lys  Gly14Glu  Lys10Asn  Gln7His | Hypothetical protein |
| 1405000  1405001  1405012  1405023 | 100  100  100  100 | C > G  A > C  A > C  G > A | 7  7  7  8 | FPSM_01279 | Gln259Glu*  Gln259Pro*  Lys263Gln  Met266Ile | Hypothetical protein |
| 1405154  1405160  1405781 | 100  100  100 | A > T  C > T  C > A | 7  8  23 | FPSM_01280 | Ser43Cys  Pro45Ser  Leu252Ile | ATP/GTP-binding protein |
| 1426533 | 83.3 | T > A | 6 | FPSM_01294 | Asn7Ile | Hypothetical protein |
| 1452344 | 98.1 | T > G | 54 | FPSM_01311 | Asn611Thr | Trypsin-like serine protease, typically periplasmic, containing C-terminal PDZ domain protein |
| 1491822 | 86.6 | A > C | 67 | FPSM_01345 | Ile450Met | ATP-dependent RNA helicase DbpA |
| 1503125 | 100 | T > G | 4 | FPSM_01358 | Glu142Ala | Histidinol-phosphate aminotransferase |
| 1561750 | 96.4 | G > A | 28 | FPSM_01408 | Glu197Lys | ATP-binding region |
| 1731007 | 83.3 | T > A | 36 | FPSM_01556 | Ile376Leu | O-antigen acetylase |
| 1761198  1762062 | 100  90 | C > G  T > C | 8  90 | FPSM_01581 | Arg358Thr  Asn70Ser | Type II restriction-modification system methylation subunit |
| 1923317  1923767 | 100  85.1 | T > C  C > T | 6  87 | FPSM_01738 | Ile794Thr  Ala944Val | PKD domain protein |
| 1958466 | 100 | T > C | 4 | FPSM_01773 | Cys13Arg | Hypothetical protein |
| 1964490 | 100 | T > A | 11 | FPSM_01781 | Phe166Ile | Transposase |
| 1980606 | 98.5 | T > C | 67 | FPSM_01797 | Val282Ala | Folylpolyglutamate synthase |
| 2265415 | 96.8 | G > C | 63 | FPSM_02051 | Val196Leu | Phospholipid-lipopolysaccharide ABC transporter |
| 2308836 | 100 | T > G | 37 | FPSM_02084 | Thr266Pro | Putative membrane spanning protein |
| 2319535  2319592 | 98.9  99.5 | G > A  C > A | 178  219 | FPSM_02089 | Pro496Ser  Asp477Tyr | DNA-directed RNA polymerase beta chain RpoB |
| 2342503 | 100 | T > A | 6 | FPSM_02111 | Thr150Ser | Lipoate-protein ligase B |
| 2344276 | 100 | G > T | 4 | FPSM_02112 | Val354Leu | Lysyl-tRNA synthetase |
| 2405414 | 90.2 | C > T | 61 | FPSM_02173 | Glu87Lys | Glycosyltransferase |
| 2438799 | 100 | T > A | 6 | FPSM_02205 | Ile288Leu | Glucose-1-phosphate thymidyltransferase |
| 2507678 | 100 | G > A | 6 | FPSM_02284 | Thr160Ile | Putative ATPase |
| 2508748 | 92.9 | A > G | 113 | FPSM_02286 | Ile279Thr | Hypothetical protein |
| 2550849  2550969 | 94.1  100 | A > G  A > C | 17  8 | FPSM_02315 | His227Arg  Asn267Thr | Putative membrane associated protein |
| 2602270 | 84 | A > C | 25 | FPSM_02358 | Trp76Gly | Hypothetical protein |
| 2697370 | 100 | C > A | 5 | FPSM_02440 | Pro25His | Hypothetical protein |
| 2697828  2697861  2697891 | 100  100  100 | G > C  C > A  G > A | 4  5  4 | FPSM_02442 | Val57Leu  Leu68Ile  Ala78Thr | Hypothetical protein |

^*^Denotes undetermined SNP call

^a^ Reference position (in bp) for reference sequence (listed under annotation field) are based on CSF 259-93 sequence from ERGO-Integrated genomics

^b^ Allele frequency refers to the proportion of sequences showing a given single-nucleotide polymorphism (SNP) at the started reference position

^c^ Shows the SNP change represents the change from nucleotide X to Y (X > Y) at the reference position

^d^ Coverage refers to the total number of sequencing reads that align to each base within the sample DNA

^e^ Predicted amino acid change for the identified SNP

^f^ Annotation shows the name and putative function for the identified gene. Annotation is presented from the CSF 259-93 sequence from ERGO-Integrated genomics

Table S3. DNA methylation motifs unique for the rifampicin passaged CSF 259-93 strain (CR).

| **DNA Motif^a^** | **Modification Type^b^** | **Motifs Detected (%)^c^** | **Number of Motifs Detected** | **Number of Motifs in Genome** | **Mean Motif Coverage** |
| --- | --- | --- | --- | --- | --- |
| CGYA**A**C | m6A | 100.00 | 1467 | 1467 | 132.0 |
| YAC**A**TC | m6A | 99.90 | 1976 | 1978 | 136.4 |
| GAAG**A**T | m6A | 99.49 | 2161 | 2172 | 135.3 |
| CGC**A**G | m6A | 98.84 | 1358 | 1374 | 138.4 |
| CTKM**A**G | m6A | 81.41 | 2496 | 3066 | 134.2 |
| GA**T**CATHH | Unknown | 66.22 | 343 | 518 | 138.2 |
| G**A**TCGMNH | Unknown | 40.94 | 235 | 574 | 138.9 |
| G**C**TGBVVNNGC | m4C | 39.80 | 119 | 299 | 141.9 |
| *Not Clustered* |  | 0.54 | 31148 | 5798830 | 141.7 |

**^a^** Degenerate base symbols represented under International Union of Pure and Applied Chemistry (IUPAC), methylated nucleotides in bold

**^b^** m6A indicates 6-methyladenosine and m4C represents 4-methylcytosine

**^c^** Percent of a methylated DNA motif’s occurrence in the genome that are methylated

Table S4. DNA methylation motifs unique for the rifampicin passaged THC 02-90 strain (TR).

| **DNA Motif^a^** | **Modification Type^b^** | **Motifs Detected (%)^c^** | **Number of Motifs Detected** | **Number of Motifs in Genome** | **Mean Motif Coverage** |
| --- | --- | --- | --- | --- | --- |
| CA**A**YNNNNNNTTYG | m6A | 99.98 | 943 | 944 | 135.2 |
| CRA**A**NNNNNNRTTG | m6A | 99.68 | 941 | 944 | 135.8 |
| CYA**A**TG | m6A | 99.88 | 3450 | 3454 | 140.6 |
| G**A**GNNNNNRTAA | m6A | 99.84 | 629 | 630 | 138.1 |
| TT**A**YNNNNNCTC | m6A | 99.84 | 629 | 630 | 140.4 |
| GGTTG**A** | m6A | 99.83 | 587 | 588 | 141.8 |
| G**A**CNNNNNNTTG | m6A | 99.47 | 744 | 748 | 140.0 |
| CA**A**NNNNNNGTC | m6A | 99.33 | 743 | 748 | 138.1 |
| CA**A**CNNNNNTG | m6A | 99.45 | 912 | 917 | 136.6 |
| C**A**NNNNNGTTG | m6A | 99.45 | 912 | 917 | 143.1 |
| GTMK**A**C | m6A | 99.4 | 666 | 670 | 140.2 |
| GRGCA**A**T | m6A | 99.27 | 683 | 688 | 134.4 |
| GNNG**A**YNNNNNNRTAG | m6A | 96.97 | 256 | 264 | 138.9 |
| CT**A**YNNNNNNRTCNNC | m6A | 95.83 | 253 | 264 | 142.3 |
| G**A**TATC | m6A | 91.87 | 689 | 750 | 141.4 |
| GA**T**CHHHH | Unknown | 49.31 | 1572 | 3188 | 145.4 |
| *Not Clustered* |  | 0.44 | 24712 | 5642000 | 145.1 |

**^a^** Degenerate base symbols represented under International Union of Pure and Applied Chemistry (IUPAC), methylated nucleotides in bold

**^b^** m6A indicates 6-methyladenosine and m4C represents 4-methylcytosine

**^c^** Percent of a methylated DNA motif’s occurrence in the genome that are methylated
